# A computational model of epithelial solute and water transport along a human nephron

**DOI:** 10.1371/journal.pcbi.1006108

**Published:** 2019-02-25

**Authors:** Anita T. Layton, Harold E. Layton

**Affiliations:** 1 Department of Mathematics, Duke University, Durham, North Carolina, United States of America; 2 Department of Biomedical Engineering, Duke University, Durham, North Carolina, United States of America; 3 Departments of Applied Mathematics and Biology, School of Pharmacy, University of Waterloo, Waterloo, Ontario, Canada; University of Michigan, UNITED STATES

## Abstract

We have developed the first computational model of solute and water transport from Bowman space to the papillary tip of the nephron of a human kidney. The nephron is represented as a tubule lined by a layer of epithelial cells, with apical and basolateral transporters that vary according to cell type. The model is formulated for steady state, and consists of a large system of coupled ordinary differential equations and algebraic equations. Model solution describes luminal fluid flow, hydrostatic pressure, luminal fluid solute concentrations, cytosolic solute concentrations, epithelial membrane potential, and transcellular and paracellular fluxes. We found that if we assume that the transporter density and permeabilities are taken to be the same between the human and rat nephrons (with the exception of a glucose transporter along the proximal tubule and the H^+^-pump along the collecting duct), the model yields segmental deliveries and urinary excretion of volume and key solutes that are consistent with human data. The model predicted that the human nephron exhibits glomerulotubular balance, such that proximal tubular Na^+^ reabsorption varies proportionally to the single-nephron glomerular filtration rate. To simulate the action of a novel diabetic treatment, we inhibited the Na^+^-glucose cotransporter 2 (SGLT2) along the proximal convoluted tubule. Simulation results predicted that the segment’s Na^+^ reabsorption decreased significantly, resulting in natriuresis and osmotic diuresis.

## Author summary

In addition to its well-known function of waste removal from the body, the kidney is also responsible for the critical regulation of the body’s salt, potassium, acid content, and blood pressure. The kidneys perform these life-sustaining task by filtering and returning to blood stream about 200 quarts of blood every 24 hours. What isn’t returned to blood stream is excreted as urine. The production of urine involves highly complex steps of secretion and reabsorption. To study these processes without employing invasive experimental procedures, we developed the first computational model of the human nephron (which is the functional unit of a kidney). The model contains detailed representation of the transport processes that take place in the epithelial cells that form the walls of the nephron. Using that model, we conducted simulations to predict how much filtered solutes and and water is transported along each individual and functionally distinct nephron segment. We conducted these simulations under normal physiological conditions, and under pharmacological conditions. The nephron model can be used as an essential component in an integrated model of kidney function in humans.

## Introduction

The parenchyma of a kidney is divided two major structures: the medulla and the outer renal cortex. In the multi-lobed human kidney, these structures take the shape of 8–18 cone-shaped renal lobes, with each resembling a uni-lobed rodent kidney, The outer region is the cortex, in which are clusters of capillaries, and convoluted segments of renal tubules. The inner region is the medulla, which further divides into the outer and inner medulla. Within the medulla one finds almost parallel arrangement of tubules and vessels [1]. Each human kidney is populated by about a million nephrons. Each nephron consists of an initial filtering component called the glomerulus and a renal tubule specialized for reabsorption and secretion. The renal tubule is the portion of the nephron in which the glomerular filtrate circulates before being excreted as urine. The functional role of the nephron is to adjust the composition of the urine so that wastes are excreted and that daily intake roughly equals urinary excretion.

The renal tubule consists of a number of segments. Given in an order consistent with fluid flow direction, the segments are: the proximal tubule, which consists of two segments, the proximal convoluted tubule (or, the S1-S2 segments) and the S3 segment; the loop of Henle, which in turn consists of a descending limb and an ascending limb; the distal convoluted tubule, the connecting tubule, and the collecting duct. Each tubular segment is lined by a single layer of epithelial cells. The ultrastructure and transport properties of the epithelial cells vary widely among different tubular segments, so that different tubular segments specialize in different roles in renal water and solute transport. Generally, the proximal tubule reabsorbs the largest fraction of the glomerular filtrate, including about two-thirds of the water and NaCl, in addition to filtered nutrients like glucose and amino acids. The thick ascending limb of the loop of Henle that follows actively pumps NaCl into the interstitium of the medulla, without water following. As a result, the fluid that reaches the distal tubule is dilute relative to blood plasma. Depending on the hydration status of the body, the collecting duct exploits this hypotonicity by either allowing (anti-diuresis) or not allowing (diuresis) water to return to general circulation via osmosis [1].

To represent physiological processes and function changes of the kidney in diseases, one may employ a useful and non-invasive approach: computational modeling. Detailed models of solute and transport have been developed for renal epithelial cells [2, 3], tubular segments [4–6], and populations of nephrons [7, 8]. All these models, and other published ones, are formulated for the rat, due to the relatively plentiful anatomic, micropuncture, and electrophysiologic data available in rodents.

It goes without saying that significant differences exist between the rat kidney and the human kidney, in terms of anatomy and hemodynamics. Consequently, while results obtained using a rat kidney model may shed insights into human kidney function, those results don’t always or entirely translate. To investigate human kidney function under physiological, pathophysiological, and pharmacological conditions, we have developed the first computational model of epithelial solute and water transport of the human nephron. Starting with our published computational model of the rat nephron [9], we incorporated anatomic and hemodynamic data from the human kidney, and we adjusted key transporter data so that the predicted urine output is consistent with known human values. Due to the relative sparsity of data on the renal transporter expression levels in humans, we identified a set of compatible transport parameters that yielded model predictions consistent with human urine and lithium clearance data.

Using the resulting model, we then explored the effects of two renal transporter inhibitors on kidney function. First, we considered an inhibitor of the sodium-glucose cotransporter 2 (SGLT2) cotransporter, which is expressed on the apical membrane of the proximal convoluted tubule and is a novel target of diabetes drugs [10]. Under normoglycemic conditions, how does the drug impact segmental Na^+^ transport and urine excretion? We also simulated inhibition of the Na^+^-K^+^-Cl^−^ cotransporter (NKCC2), which is expressed on the apical membrane of the thick ascending limbs of the loops of Henle and aids in the active transport of Na^+^, K^+^, and Cl^−^ into the cell. How substantial are the compound’s diuretic, natriuretic, and kaliuretic effects?

## Materials and methods

We have developed an epithelial cell-based model of a superficial nephron of a human kidney, extending from Bowman’s capsule to the papillary tip. The model is based on previously-applied models of the rat nephron [9, 11, 12]. The model nephron is represented as a tubule lined by a layer of epithelial cells, with apical and basolateral transporters that vary according to cell type. See figure 1 in Ref. [9] for a schematic diagram. The model assumes that the connecting tubules coalesce successively within the cortex, resulting in a ratio of loop-to-cortical collecting duct of 10:1 [13]. In the inner medulla, the collecting ducts again coalesce successively.

The model accounts for 15 solutes: Na^+^, K^+^, Cl^−^, ${\text{HCO}}_{3}^{-}$, H_2_CO_3_, CO_2_, NH_3_, ${\text{NH}}_{4}^{+}$, ${\text{HPO}}_{4}^{2-}$, ${\text{H}}_{2}{\text{PO}}_{4}^{-}$, H^+^, ${\text{HCO}}_{2}^{-}$, H_2_CO_2_, urea, and glucose. The model is formulated for steady state and consists of a large system of coupled ordinary differential equations and algebraic equations (see model equations below). Model solution predicts luminal fluid flow, hydrostatic pressure, luminal fluid solute concentrations, cytosolic solute concentrations, epithelial membrane potential, and transcellular and paracellular fluxes.

### Model equations

#### Conservation in the cellular and paracellular compartments

At steady state, water conservation in the cellular and paracellular (i.e., lateral) compartments (denoted by subscripts ‘C’ and ‘P’, respectively) of tubular segment *i* is given by

$$\begin{array}{cc} J_{v,\text{LC}}^{i}+J_{v,\text{BC}}^{i}+J_{v,\text{PC}}^{i} & =0 \end{array}$$ (1)

$$\begin{array}{cc} J_{v,\text{LP}}^{i}+J_{v,\text{BP}}^{i}+J_{v,\text{CP}}^{i} & =0 \end{array}$$ (2)

where the subscripts ‘L’ and ‘B’ denote lumen and blood (i.e., interstitium), respectively. In the above notations, water flux $J_{v,ab}^{i}$ is taken positive from compartment *a* to *b*.

Conservation of non-reacting solute *k* is given by

$$\begin{array}{cc} J_{k,\text{LC}}^{i}+J_{k,\text{BC}}^{i}+J_{k,\text{PC}}^{i} & =0 \end{array}$$ (3)

$$\begin{array}{cc} J_{k,\text{LP}}^{i}+J_{k,\text{BP}}^{i}+J_{k,\text{CP}}^{i} & =0 \end{array}$$ (4)

where $J_{k,ab}^{i}$ denotes the transmembrane flux of solute *k* from compartment *a* to *b*.

For the reacting solutes, conservation is applied to the total buffers:

$$\begin{array}{cc} {\hat{J}}_{{\text{CO}}_{2},m}^{i}+{\hat{J}}_{{\text{HCO}}_{3}^{-},m}^{i}+{\hat{J}}_{H_{2}{\text{CO}}_{3},m}^{i} & =0 \end{array}$$ (5)

$$\begin{array}{cc} {\hat{J}}_{{\text{HPO}}_{4}^{2-},m}^{i}+{\hat{J}}_{H_{2}{\text{PO}}_{4}^{-},m}^{i} & =0 \end{array}$$ (6)

$$\begin{array}{cc} {\hat{J}}_{{\text{NH}}_{3},m}^{i}+{\hat{J}}_{{\text{NH}}_{4}^{+},m}^{i} & =0 \end{array}$$ (7)

$$\begin{array}{cc} {\hat{J}}_{{\text{HCO}}_{2}^{-},m}^{i}+{\hat{J}}_{H_{2}{\text{CO}}_{2},m}^{i} & =0 \end{array}$$ (8)

where *m* corresponds to ‘C’ or ‘P’. ${\hat{J}}_{k,m}^{i}$ denotes the net flux of solute *k* into compartment *m*; specifically, ${\hat{J}}_{k,C}^{i}\equiv J_{k,\text{LC}}^{i}+J_{k,\text{BC}}^{i}+J_{k,\text{PC}}^{i}$ and ${\hat{J}}_{k,P}^{i}\equiv J_{k,\text{LP}}^{i}+J_{k,\text{BP}}^{i}+J_{k,\text{CP}}^{i}$.

The buffer pairs are assumed to be in equilibrium:

$$\begin{array}{c} {\text{pH}}_{m}^{i}={\text{pK}}_{A}-\log\frac{C_{A,m}^{i}}{C_{B,m}^{i}} \end{array}$$ (9)

where $C_{k,m}^{i}$ denotes the concentration of solute *k* in compartment *m*. The buffer pairs (A,B) are $\left({\text{HCO}}_{3}^{-},{\text{H}}_{2}{\text{CO}}_{3}\right)$, $\left({\text{HPO}}_{4}^{2-},{\text{H}}_{2}{\text{PO}}_{4}^{-}\right)$, $\left({\text{NH}}_{3},{\text{NH}}_{4}^{+}\right)$, and $\left({\text{HCO}}_{2}^{-},{\text{H}}_{2}{\text{CO}}_{2}\right)$. The pH of compartment *m* is given by conservation of hydrogen ion:

$$\begin{array}{c} \sum_{k}{\hat{J}}_{k,m}^{i}=0 \end{array}$$ (10)

where the summation index *k* is applied over the solutes H^+^, ${\text{NH}}_{4}^{+}$, ${\text{H}}_{2}{\text{PO}}_{4}^{-}$, H_2_CO_3_, and H_2_CO_2_.

#### Conservation in the lumen

Within the lumen of a non-coalescing tubule *i*, conservation of water and non-reacting solutes at steady state is given by

$$\begin{array}{cc} & \frac{dQ^{i}}{dx}={\hat{J}}_{v,L}^{i} \end{array}$$ (11)

$$\begin{array}{cc} & \frac{d}{dx}(Q^{i}C_{k,L}^{i})={\hat{J}}_{k,L}^{i} \end{array}$$ (12)

where *Q*^*i*^ denotes volume flow (per tubule). ${\hat{J}}_{v,L}^{i}\equiv J_{v,LC}^{i}+J_{v,LP}^{i}$ denotes overall (transcellular and paracellular) water flux and ${\hat{J}}_{k,L}$ denotes the analogous solute flux. *x* is the axial position.

For the reacting solutes, conservation is applied to the total buffers:

$$\begin{array}{cc} \frac{d}{dx} & (Q^{i}C_{{\text{CO}}_{2},L}^{i}+Q^{i}C_{{\text{HCO}}_{3},L}^{i}+Q^{i}C_{H_{2}{\text{CO}}_{3},L}^{i})={\hat{J}}_{{\text{CO}}_{2},L}^{i}+{\hat{J}}_{{\text{HCO}}_{3},L}^{i}+{\hat{J}}_{H_{2}{\text{CO}}_{3},L}^{i} \end{array}$$ (13)

$$\begin{array}{cc} \frac{d}{dx} & (Q^{i}C_{{\text{HPO}}_{4},L}^{i}+Q^{i}C_{H_{2}{\text{PO}}_{4},L}^{i})={\hat{J}}_{{\text{HPO}}_{4},L}^{i}+{\hat{J}}_{H_{2}{\text{PO}}_{4},L}^{i} \end{array}$$ (14)

$$\begin{array}{cc} \frac{d}{dx} & (Q^{i}C_{{\text{NH}}_{3},L}^{i}+Q^{i}C_{{\text{NH}}_{4},L}^{i})={\hat{J}}_{{\text{NH}}_{3},L}^{i}+{\hat{J}}_{{\text{NH}}_{4},L}^{i} \end{array}$$ (15)

$$\begin{array}{cc} \frac{d}{dx} & (Q^{i}C_{{\text{HCO}}_{2},L}^{i}+Q^{i}C_{H_{2}{\text{CO}}_{2},L}^{i})={\hat{J}}_{{\text{HCO}}_{2},L}^{i}+{\hat{J}}_{H_{2}{\text{CO}}_{2},L}^{i} \end{array}$$ (16)

The buffer pairs are assumed to be in equilibrium (Eq 9).

For a coalescing tubule, i.e., the connecting tubule or inner-medullary collecting duct (*i* = CNT or IMCD), the equations are modified by scaling water and solute flows by the tubule population *ω*^*i*^. For example, conservation of luminal fluid and non-reacting solutes is given by

$$\begin{array}{cc} & \frac{d}{dx}(\omega^{i}Q^{i})={\hat{J}}_{v,L}^{i} \end{array}$$ (17)

$$\begin{array}{cc} & \frac{d}{dx}(\omega^{i}Q^{i}C_{k,L}^{i})={\hat{J}}_{k,L}^{i} \end{array}$$ (18)

Eqs 13–16 are modified similarly. The convergence of the connecting tubules is described by *ω*^CNT^, which denotes the fraction of connecting tubules remaining at coordinate *x*^CNT^:

$$\begin{array}{c} \omega^{\text{CNT}}\left(x^{\text{CNT}}\right)=2^{-3.32\;x^{\text{CNT}}/L^{\text{CNT}}} \end{array}$$ (19)

where *L*^CNT^ denotes the connecting tubule length and *x*^CNT^ denotes the distance from the connecting tubule entrance. Eq 19 is formulated such that at the end of the connecting tubule, 10% of the population remains (i.e., *ω*^CNT^(*L*^CNT^) = 0.1). These remaining connecting tubules converge into the cortical collecting duct.

The inner-medullary collecting ducts coalesce in a similar manner. Let *ω*^IMCD^(*x*) denote the number of inner-medullary collecting ducts per nephron:

$$\begin{array}{c} \omega^{\text{IMCD}}\left(x^{\text{IMCD}}\right)=0.1\times (1-0.95{\left(\frac{x^{\text{IMCD}}}{L^{\text{IMCD}}}\right)}^{2})\exp^{-2.75\;x^{\text{IMCD}}/L^{\text{IMCD}}} \end{array}$$ (20)

where *L*^IMCD^ denotes the inner-medullary collecting duct length and *x*^IMCD^ denotes the distance from its entrance. Eq 20 is formulated to represents 8 convergences of the collecting ducts along the inner medulla.

As noted above, the convergence of the connecting tubules results in a 10:1 loop-to-cortical collecting duct (CCD) ratio. This configuration yields the following cortical collecting duct inflow conditions. For luminal fluid and non-reacting solutes:

$$\begin{array}{cc} & Q^{\text{CCD}}=10\times Q^{\text{CNT}} \end{array}$$ (21)

$$\begin{array}{cc} & Q^{\text{CCD}}C_{k,L}^{\text{CCD}}=10\times Q^{\text{CNT}}C_{k,L}^{\text{CNT}} \end{array}$$ (22)

For the reacting solutes, total buffer is conserved:

$$\begin{array}{c} Q^{\text{CCD}}\sum_{k}C_{k,L}^{\text{CCD}}=10\times Q^{\text{CNT}}\sum_{k}C_{k,L}^{\text{CNT}} \end{array}$$ (23)

The summation over *k* is applied to each buffer group. For example, one instance of Eq 23 is applied over CO_2_, ${\text{HCO}}_{3}^{-}$, and H_2_CO_3_, another over ${\text{HPO}}_{4}^{2-}$, and ${\text{H}}_{2}{\text{PO}}_{4}^{-}$, and so forth. Each buffer pair is assumed to be in equilibrium and satisfies Eq 9. Conservation of hydrogen ion is also applied:

$$\begin{array}{c} Q^{\text{CCD}}\sum_{k}C_{k,L}^{\text{CCD}}=10\times Q^{\text{CNT}}\sum_{k}C_{k,L}^{\text{CNT}} \end{array}$$ (24)

where the summation index *k* is applied over the solutes H^+^, NH_4_, H_2_PO_4_, H_2_CO_3_, and H_2_CO_2_.

#### Tubular flow, fluid pressure, and flow-dependent transport

Tubular fluid flow is described by the pressure-driven Poiseuille flow. The hydrostatic pressure in the lumen of tubule *i*, *P*^*i*^, is related to volume flow *Q*_*i*_ (per tubule) and luminal radius *r*_*i*_ by

$$\begin{array}{c} \frac{dP^{i}}{dx}=-\frac{8\mu Q^{i}}{\pi {\left(r^{i}\right)}^{4}} \end{array}$$ (25)

where *μ* is the luminal fluid viscosity (taken as 6.4 × 10^−6^ mmHg⋅s^−1^).

Proximal tubule reabsorption varies proportionally to SNGFR [14]. To model flow-dependent transepithelial transport, we follow the approach of Weinstein et al. [7]. Specifically, the proximal tubule is assumed to be compliant, with luminal radius given by

$$\begin{array}{c} r^{\text{PT}}=r_{0}^{\text{PT}}(1+\nu^{\text{PT}}(P^{\text{PT}}-P_{0}^{\text{PT}})) \end{array}$$ (26)

where the reference radius $r_{0}^{\text{PT}}$ is taken as 14 *μ*m, the reference pressure $P_{0}^{\text{PT}}$ is taken as 20 mmHg, and *ν*^PT^, which characterizes tubular compliance, is set to 0.03.

To account for the modulation of transporter density by luminal flow, we determine the microvillous torque as

$$\begin{array}{c} \tau^{\text{PT}}=\frac{8\mu Q^{\text{PT}}l^{\text{PT},\text{mv}}}{{\left(r^{\text{PT}}\right)}^{2}}(1+\frac{l^{\text{PT},\text{mv}}+\delta^{\text{PT},\text{mv}}}{r^{\text{PT}}}+\frac{{\left(l^{\text{PT},\text{mv}}\right)}^{2}}{2{\left(r^{\text{PT}}\right)}^{2}}) \end{array}$$ (27)

where *l*^PT,mv^ = 2.5 *μ*m is the microvillous length, and *δ*^PT,mv^ = 0.15 *μ*m denotes the height above the microvillous tip where drag is considered [15]. The density of apical and basolateral transporters in proximal tubule cells is scaled by:

$$\begin{array}{c} 1+s(\frac{\tau^{\text{PT}}}{\tau_{0}^{\text{PT}}}-1) \end{array}$$ (28)

where the reference torque $\tau_{0}^{\text{PT}}$ is evaluated at reference flow set to the inflow of the proximal tubule (100 nl/min, see below). The scaling factor *s* is taken to be 1.5 for the cortical segment (i.e., the proximal convoluted tubule or S1-S2 segment) and 0.75 for the medullary segment (i.e., the proximal straight tubule or the S3 segment).

Nephron segments downstream of the proximal tubule are represented by rigid tubes. Tubular transport is assumed to be independent of flow.

#### Flux calculations

Volume fluxes are calculated using the Kedem-Katchalsky equation [16]:

$$\begin{array}{c} J_{v,ab}^{i}=A_{ab}^{i}L_{p,ab}^{i}(\sigma_{ab}^{i}\Delta \pi_{ab}^{i}+\Delta P_{ab}^{i}) \end{array}$$ (29)

where $A_{ab}^{i}$ denotes the area separating compartments *a* and *b*, $L_{p,ab}^{i}$ is the hydraulic permeability, $\Delta \pi_{ab}^{i}\equiv RT\sum \Delta C_{ab}^{i}$ is the osmotic pressure gradient, *RT* is the product of the gas constant and thermodynamic temperature, $\sum \Delta C_{ab}^{i}$ is the osmolalty difference, $\sigma_{ab}^{i}$ is the reflective coefficient, and $\Delta P_{ab}^{i}$ denotes the hydrostatic pressure gradient.

Ions may be driven across a membrane by an electrochemical gradient across ion channels. Those electrodiffusive fluxes are described using the Goldman-Hodgkin-Katz current equation:

$$\begin{array}{c} J_{k,ab}^{i}=A_{ab}^{i}\rho_{k,ab}^{i}\frac{z_{k}F\Delta V_{ab}^{i}}{RT}\frac{C_{k,a}^{i}-C_{k,b}^{i}\exp\left(-z_{k}F\Delta V_{ab}^{i}/RT\right)}{1-\exp(-z_{k}F\Delta V_{ab}^{i}/RT)} \end{array}$$ (30)

where $\rho_{k,ab}^{i}$ denotes membrane permeability to solute *k*, $\Delta V_{ab}^{i}$ is the electrical potential gradient, *z*_*k*_ is the valance, and *F* is the Faraday’s constant. For an uncharged solute, the above equation reduces to:

$$\begin{array}{c} J_{k,ab}^{i}=A_{ab}^{i}\rho_{k,ab}^{i}(C_{k,a}^{i}-C_{k,b}^{i}) \end{array}$$ (31)

Transmembrane solute flux may include additional components, depending on the solute, such as coupled transport across cotransporters and/or exchangers and primary active transport across ATP-driven pumps [17]. The impacts of inhibition SGLT2 and NKCC2 are explored in this study. Thus, we describe below how these cotransporters are modeled.

**Fig 1:**
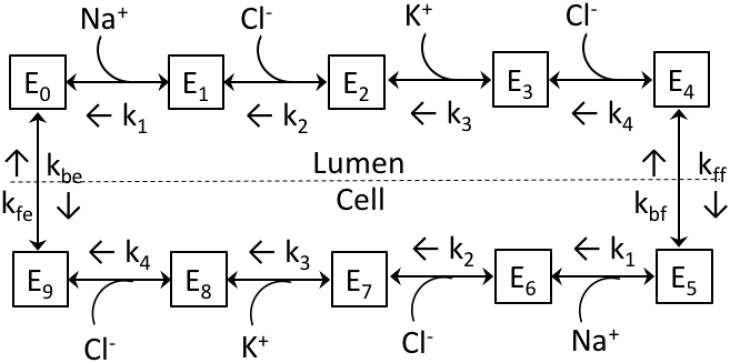
Kinetic model of the renal NKCC2 cotransporter. *k*_*j*_’s, off-binding rate constants, on-binding rate constants not shown. *k*_ff_, *k*_bf_, *k*_fe_, and *k*_be_, translocation constant rates; *E*_*j*_, contransporter density of state *j*.

The NKCC2 cotransporter is expressed on the apical membrane of the thick ascending limb. The NKCC2 model, illustrated in Fig 1, is based on our published thick ascending limb model [5], which is in turn based on Ref. [18]. The model assumes sequential binding of the ions following the order: Na^+^, Cl^−^, K^+^, and Cl^−^. The transporter undergoes a transformational change, and the ions are assumed to be released into the cell in the same order as they bound in the lumen. The unloaded transporter is then transformed into a state where it is available for luminal ion binding. All reactions are assumed to be reversible. A set of model equations, which assume steady state and apply the principle of detailed balance, can be found in the Appendix of Ref. [18].

The kinetic behavior of SGLT2 is modeled based upon the sodium-alanine cotransporter model [19], SGLT2-mediated flux is calculated as

$$\begin{array}{cc} J_{\text{glu}}^{\text{SGLT}2} & =\frac{X_{\text{SGLT}2}k_{u}^{f}}{\Phi }(C_{\text{glu}}^{\text{lum}}C_{{\text{Na}}^{+}}^{\text{lum}}\exp\left(\zeta \right)-C_{\text{glu}}^{\text{cyt}}C_{{\text{Na}}^{+}}^{\text{cyt}}) \end{array}$$ (32)

$$\begin{array}{cc} J_{{\text{Na}}^{+}}^{\text{SGLT}2} & =J_{\text{glu}}^{\text{SGLT}2} \end{array}$$ (33)

where

$$\begin{array}{c} \zeta =F\left(\Psi_{\text{lum}}-\Psi_{\text{cyt}}\right)/\left(RT\right) \end{array}$$ (34)

$$\begin{array}{c} n^{\text{lum}}=\frac{C_{{\text{Na}}^{+}}^{\text{lum}}}{K_{m,{\text{Na}}^{+}}^{\text{SGLT}2}},\;n^{\text{cyt}}=\frac{C_{{\text{Na}}^{+}}^{\text{cyt}}}{K_{m,{\text{Na}}^{+}}^{\text{SGLT}2}},\;g^{\text{lum}}=\frac{C_{\text{glu}}^{\text{lum}}}{K_{m,\text{glu}}^{\text{SGLT}2}},\;g^{\text{cyt}}=\frac{C_{\text{glu}}^{\text{cyt}}}{K_{m,\text{glu}}^{\text{SGLT}2}} \end{array}$$

$$\begin{array}{c} \Phi =\left(1+n^{\text{lum}}+g^{\text{lum}}+n^{\text{lum}}g^{\text{lum}}\right)\left(1+n^{\text{cyt}}g^{\text{cyt}}\right)+\left(1+n^{\text{cyt}}+g^{\text{cyt}}+n^{\text{cyt}}g^{\text{cyt}}\right)\left(1+n^{\text{lum}}g^{\text{lum}}\exp\left(\zeta \right)\right) \end{array}$$ (35)

*X*_SGLT2_ characterizes the density of SGLT2 transporters, $k_{u}^{f}$ is the forward translocation rate of the unloaded carrier, $C_{\text{glu}}^{\text{lum}}$ and $C_{{\text{Na}}^{+}}^{\text{lum}}$ respectively denote the luminal concentration of glucose and Na^+^, $C_{\text{glu}}^{\text{cyt}}$ and $C_{{\text{Na}}^{+}}^{\text{cyt}}$ denote the cytosolic concentration of glucose and Na^+^, and *Ψ*_lum_ and *Ψ*_cyt_ represent the electric potential in the lumen and cytosol. Lastly, $K_{m,\text{glu}}^{\text{SGLT}2}$ and $K_{m,{\text{Na}}^{+}}^{\text{SGLT}2}$ respectively denote the binding affinity of SGLT2 to glucose and Na^+^. Eq 32 posits a simultaneous mechanism for the transport of Na^+^ and glucose. It also assumes that (a) the binding affinities to a given ion on the luminal and cytosolic sides of the membrane are the same, (b) the forward $\left(k_{u}^{f}\right)$ and backward $\left(k_{u}^{b}\right)$ translocation rates of the unloaded carrier, and the backward translocation rate of the fully loaded carrier $\left(k_{l}^{b}\right)$, are all equal, and (c) the forward translocation rate of the fully loaded carrier $\left(k_{l}^{f}\right)$ is given by $\left(k_{u}^{f}k_{l}^{b}/k_{u}^{b}\right)\cdot \exp\left(\zeta \right)$, so as to satisfy thermodynamics constraints (i.e., the principle of microscopic reversibility).

### Model parameters

The typical volume of a kidney from a 340-g rat is ∼1.32 ml, with the cortex, outer medulla, and inner medulla occupying 63, 34, and 3%, respectively, of that volume [20]. The volume of a kidney from a 30–40-year old adult human was reported to be 285 ml, with the cortex, outer medulla, and inner medulla occupying 63, 27, and 10%, respectively, of that volume [21]. Thus, the human kidney is >200 times bigger than a rat kidney. That large difference in volume can be attributed, in part but not solely, to the ∼30-times larger glomerulus population in a human kidney (1 million versus 36,000).

The model medulla is taken to be 17 mm [22], with the outer and inner medulla taken to be 5 and 12 mm, respectively, based on Ref. [23] and personal communication from Tom Pannabecker. Tubular length and luminal diameter values are given in Table 1. Tubular lengths were gleaned from Refs. [22–24]. Tubular luminal diameters are estimated from renal biopsy studies of health individuals [25–28].

**Table 1:** Nephron segment lengths and luminal diameters.

Segment	Length (cn)	Diameter (*μ*m)
Proximal tubule	1.7	37
Descending limb	0.32	26
Medullary thick ascending limb	0.5	26
Cortical thick ascending limb	0.5	26
Distal convoluted tubule	0.2	20
Connecting tubule	0.4	24
Cortical connecting duct	0.4	45
Outer-medullary connecting duct	0.5	45
Inner-medullary connecting duct	1.2	50

The composition of the interstitial fluid in the cortex at the boundary between the outer and inner medulla, and at the papillary tip is specified in Table 2. We assume that interstitial concentrations vary linearly between the cortico-medullary junction and the inner-outer medullary boundary, and between the inner-outer medullary boundary and the papillary tip. The interstitial fluid in the cortex is taken to be homogeneous except for ammonia (see Ref. [9]). Tubular fluid concentrations at the proximal tubule inlet are equal to those in the cortical interstitium, except for the absence of protein.

**Table 2:** Interstitial concentrations (in mM, unless stated otherwise).

Solute	Cortex	OM-IM boundary	Papillary tip
Na^+^	140.0	299.0	239.0
K^+^	4.0	8.0	15.0
Cl^−^	100.0	280.4	236.5
${\text{HCO}}_{3}^{-}$	25.0	25.0	25.0
${\text{H}}_{2}{\text{CO}}_{3}^{-}$	4.41 × 10^−3^	4.41 × 10^−3^	4.41 × 10^−3^
CO_2_	1.45	1.45	1.45
${\text{HPO}}_{4}^{2-}+{\text{H}}_{2}{\text{PO}}_{4}^{-}$	1.45	1.45	1.45
urea	5.0	60.0	200
${\text{NH}}_{3}+{\text{NH}}_{4}^{+}$	1.0	3.9	8.95
${\text{HCO}}_{2}^{-}+{\text{H}}_{2}{\text{CO}}_{2}$	1.0	1.0	1.0
glucose	5.0	6.25	7.5
protein	2.0	2.0	2.0
pH (dimensionless)	7.4	7.4	7.3
osmolality (mosm/(kg H_2_O))	285.9	688.5	737.9

OM-IM boundary: at the junction between the outer and inner medulla.

The model represents only a superficial nephron because in human, the vast majority of the nephrons (∼85%) are superficial [24]. Normal human glomerular filtration rate (GFR) falls within the range of 90–120 ml/min/1.73m^2^ [29]. Single-nephron GFR (SNGFR) is taken to be 100 nl/min, assuming 1 million nephrons [30]. Proximal tubule inflow fluid pressure is taken to be 21 mmHg [31]. Unless otherwise specified, model parameters specifying transporter density and water and solute permeability are taken to be the corresponding rat values [5, 9, 17, 32]; see below for exceptions.

## Results

*To what extent can functional differences between the rat and human kidneys be accounted for by their anatomical and hemodynamic differences alone?* To answer that question, we conducted model simulations after incorporating human tubular lengths and luminal diameters (Table 1), loop-to-cortical collecting duct ratio (10:1), and SNGFR (100 nl/min) into our published rat nephron model [9]. Additionally, the interstitial concentration profiles, which exhibit only minor adjustments from the rat values (compare Table 2 with Table 3 in Ref. [9]), were incorporated. Transporter densities and permeabilities were set to the values of the rat nephron model [9]. Due to the longer length and larger surface area of the human nephron segments, total transporter protein amounts are larger in the human model.

Table 3, column “GFR & dimension,” shows urine composition obtained with human kidney anatomy and GFR incorporated. The model predicted a urine flow of 1.2 L/24h per person, which falls within the typical range of 0.8–2 L/24h. While urine composition is consistent with typical human values [33, 34], urine pH was predicted to be 5.1, which is substantially more acidic than expected [33]. Also, the model predicted that essentially all filtered glucose is reabsorbed by the proximal convoluted tubule (not shown), a result that is inconsistent with the expectation that the S3 segment mediates ∼10% of glucose transport.

**Table 3:** Urine flow (per kidney) and composition, obtained under differing conditions. *Model with human GFR and anatomical parameters, but rat transporter densities and permeabilities.

	Baseline	Measured	GFR & dimensions*	SGLT2 inhibition	NKCC2 inhibition
Flow (ml/min)	0.62	0.55–1.4 [33]	0.41	1.5	1.7
Na^+^ (mM)	73	51–191 [34]	83	44	47
K^+^ (mM)	72	19–67 [34]	52	52	62
Cl^−^ (mM)	69	53–237 [34]	52	69	87
Urea (mM)	280	155–388 [34]	260	198	111
pH	6.2	6.2 [33]	5.1	6.4	6.3

To reconcile the above discrepancies, we hypothesize that *human proximal tubule expresses lower SGLT2 density compared to rat*, and we lowered the SGLT2 density along the S1-S2 segment by 25%. To increase urine pH, we hypothesize that *the human collecting duct has lower H^+^-ATPase and H^+^-K^+^-ATPase densities relative to rat* and we reduced H^+^-ATPase and H^+^-K^+^-ATPase densities along these segments by 50%. These revised transporter parameters, together with the human anatomical and hemodynamic values, form the *baseline model parameters set*.

**Fig 2:**
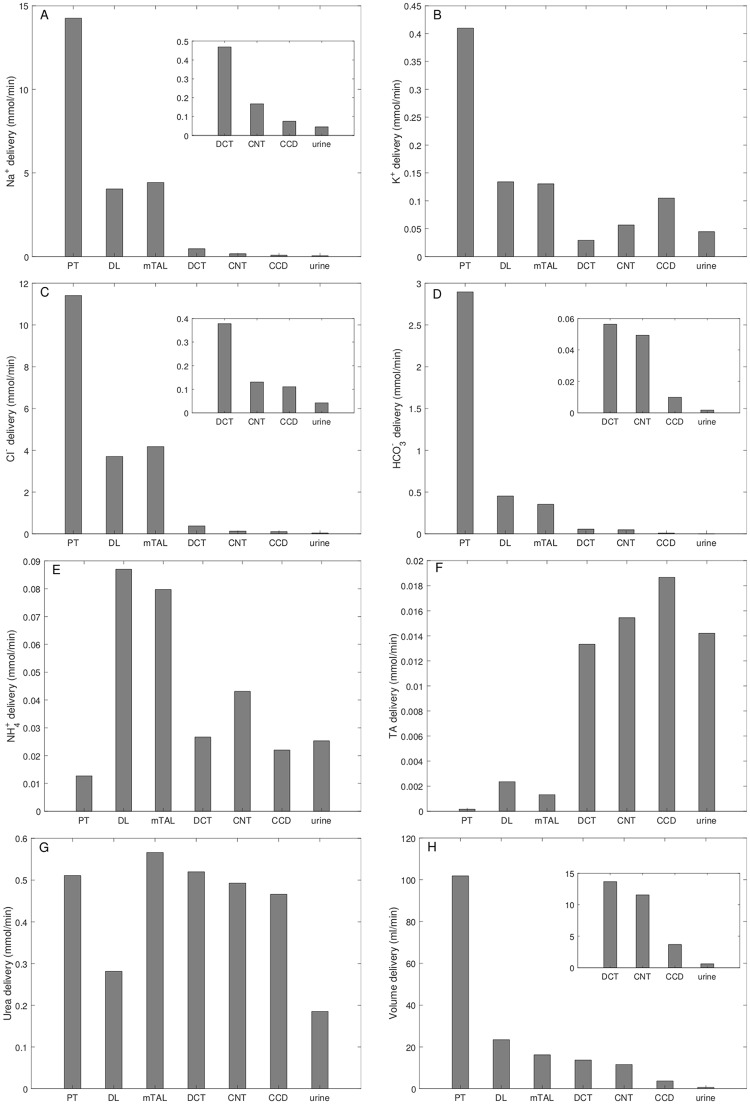
Total delivery of key solutes (*A–G*) and fluid (*H*) to the beginning of individual nephron segments, given per kidney. Model predicts that the majority of key solutes and volume are reabsorbed by the proximal tubule (PT). The distal tubular segments are responsible for the secretion of K^+^ and acid species. DL, descending limb; mTAL, medullary thick ascending limb; DCT, distal convoluted tubule; CNT, connecting duct; CCD, cortical collecting duct. TA, titratable acid. Insets reproduce distal segment and urine values.

With the baseline parameter sets, the model predicted a urine flow of 1.2 ml/min, or 1.8 L/24h; see Table 3, column “Baseline.” Urinary Na^+^, K^+^, and Cl^−^ excretion were predicted to be 91, 89, and 85 *μ*mol/min per person. The predicted delivery of key solutes (Na^+^, K^+^, Cl^+^, ${\text{HCO}}_{3}^{-}$, ${\text{NH}}_{4}^{+}$, TA, and urea) and fluid to the inlets of individual nephron segments; these results are shown in Fig 2. These values are given per kidney. The concentration of titratable acid (TA) is evaluated from [H_2_PO_4_] and [HPO_4_]:

$$\begin{array}{c} \left[\text{TA}\right]=\frac{10^{\left(7.4-6.8\right)}\left[H_{2}{\text{PO}}_{4}\right]-\left[{\text{HPO}}_{4}\right]}{1+10^{\left(7.4-6.8\right)}} \end{array}$$ (36)

Collecting duct outflow pressure was predicted to be 5.1 mmHg.

The model predicted that the proximal tubule reabsorbed 72% of the filtered Na^+^, primarily via the Na^+^/H^+^-exchanger 3 (NHE3; apical membrane) and Na^+^/K^+^-ATPase (basolateral membrane); see Fig 2A. 61% and 39% of the proximal tubular Na^+^ reabsorption occured transcellularly and paracellularly, respectively. That Na^+^ transport was accompanied by the reabsorption of 68 and 84% of filtered Cl^−^ and ${\text{HCO}}_{3}^{-}$, respectively; see Fig 2C and 2D. The majority of the remaining Na^+^ and Cl^−^ was reabsorbed along the thick ascending limbs, resulting in urine excretion fractions of ∼0.3% for both Na^+^ and Cl^−^. Similarly, the model predicted that 67% of the filtered K^+^ was reabsorbed along the proximal tubule; again, the majority of the remaining K^+^ is reabsorbed along the thick ascending limb. Along the distal convoluted tubule and connecting tubule, Na^+^ transport generated a favorable electrochemical gradient for K^+^ secretion. Thus, these segments were predicted to vigorously secrete K^+^ (see Fig 2B). Consequently, urinary K^+^ excretion corresponds to 11% of the filtered load. NHE3 mediated the secretion of a substantial amount of ${\text{NH}}_{4}^{+}$ into the proximal tubule lumen, via its competition with H^+^ (see Fig 2E).

**Fig 3:**
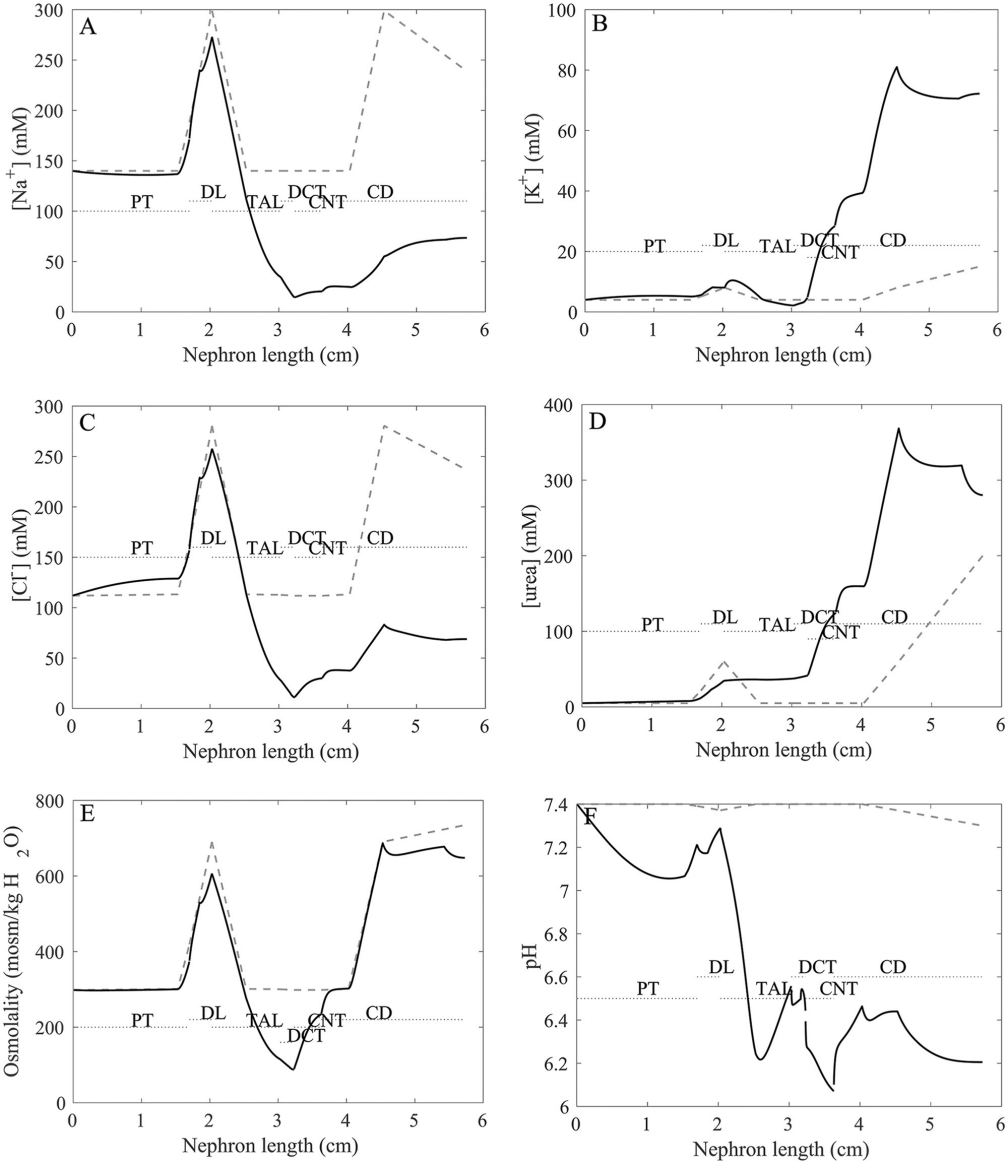
Profiles of tubular fluid solute concentrations (*A–D*), osmolality (*E*), and pH (*F*). Solid black lines, superficial nephron. Solid line, luminal fluid solute concentrations, pH, and osmolality. Dashed lines, interstitial values. PT, proximal tubule; DL, descending limb; LDL/LAL, thin descending/ascending limb; TAL, thick ascending limb; DCT, distal convoluted tubule; CNT, connecting duct; CD, collecting duct.

Tubular and interstitial fluid concentration of key solutes, pH, and osmolality are shown in Fig 3 (panels A–F). Recall the interstitial concentration of key solutes was taken to increase along the medullary axis for a kidney in anti-diuretic state. Fluid osmolality of water-permeable tubular segments closely tracks interstitial fluid osmolality (Fig 3E). Tubular urea concentration increases along flow direction (Fig 3D) due to water reabsorption. Luminal fluid is significantly acidified along the thick ascending limb (Fig 3F), a process that is also reflected in the increase in titratable acid flow along that segment (Fig 2F). The model predicted a urine osmolality of 648 osmol/(kg H_2_O), and urine [Na^+^], [K^+^], [Cl^−^], and [urea] of 73, 72, 69, and 280 mM, respectively (Table 3, column “Baseline”); electroneutrality is maintained by other ions, such as phosphate and bicarbonate. Urine pH was predicted to be 6.2. These predictions are consistent with values reported in humans [33].

Taken together, under baseline conditions, the model proximal tubule and the thick ascending limb are the primary segments responsible for the reabsorption of filtered Na^+^ and K^+^, that the distal tubular segments secrete a substantial amount of K^+^, and that the thick ascending limb and, to a lesser extent, the inner-medullary collecting duct acidify the luminal fluid.

### Varying SNGFR

**Fig 4:**
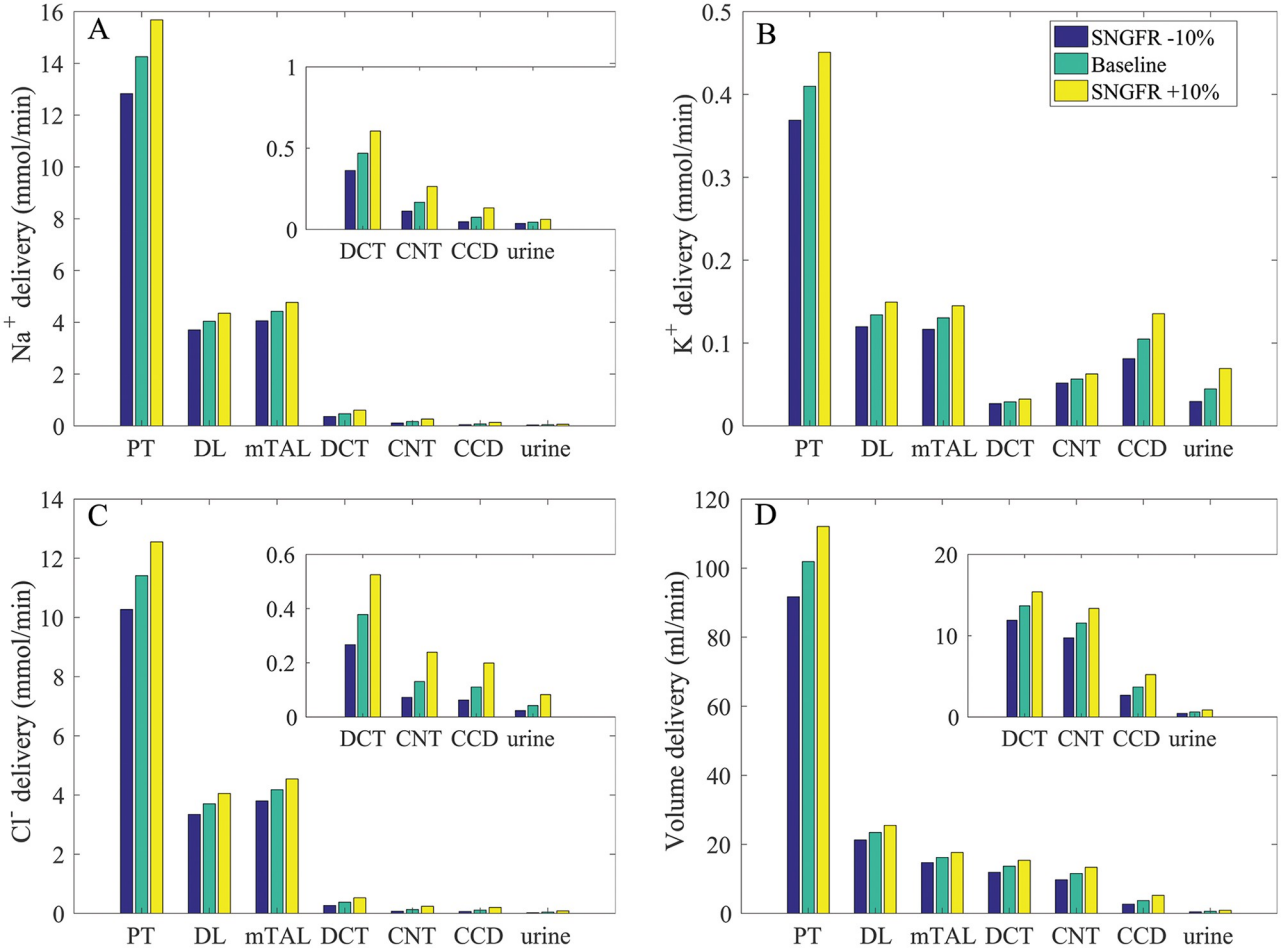
Impact of varying SNGFR on segmental delivery of Na^+^, K^+^, Cl^−^, and fluid, given per kidney. A higher SNGFR enhances solute and volume deliveries, with the effect proportionally larger in distal segments. Notations are analogous to Fig 2. Insets reproduce distal segment and urine values.

**Fig 5:**
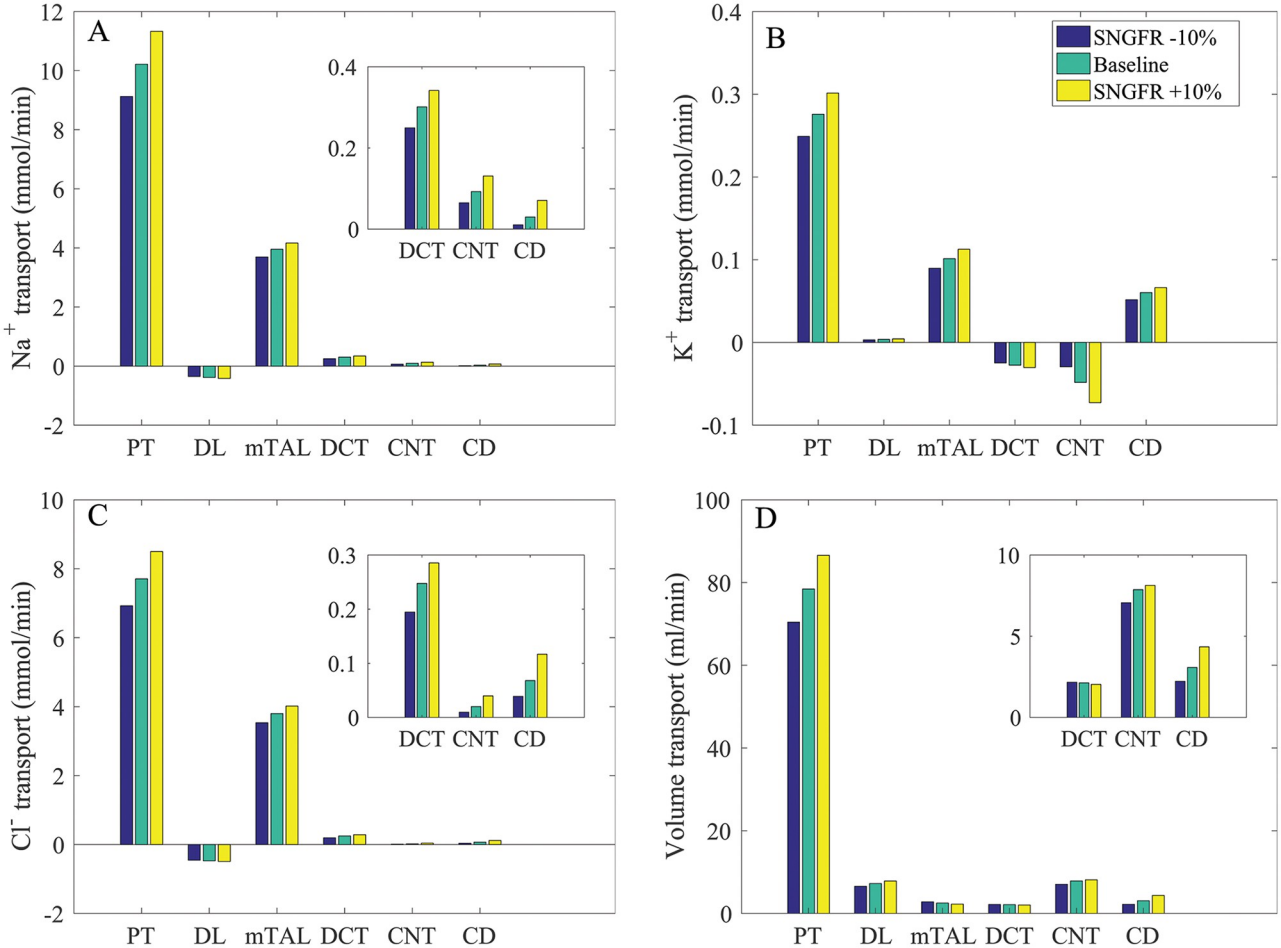
Impact of varying SNGFR on predicted transport of Na^+^, K^+^, Cl^−^, and fluid, given per kidney. Positive values denote reabsorption. Notably, a 10% increase in SNGFR doubles K^+^ secretion along the connecting tubules. Insets reproduce distal segment and urine values.

The proximal tubule of the rat kidney is known to exhibit glomerulotubular balance [35], such that tubular Na^+^ reabsorption changes in proportion to SNGFR to maintain approximately the same fractional reabsorption. Glomerulotubular balance has not been demonstrated experimentally in humans. To assess the relationship between segmental transport to SNGFR in the human nephron, we conducted simulations where SNGFR was varied by ±10% of baseline values. We assumed that changes in SNGFR are due to changes in renal blood flow, as opposed to changes in filtration fraction which are linked to changes in peritubular oncotic pressure. Thus, as was done in a previous rat modeling study [11], we assumed no change in proximal tubule transport expression levels, except in response to changes in torque. (Model proximal tubule transepithelial transport is assumed to be flow dependent. Details can be found in Ref. [11]). The predicted Na^+^, K^+^, Cl^−^, and volume deliveries to individual segments under differing SNGFR are shown in Fig 4; the corresponding solute and fluid transport are shown in Fig 5.

When SNGFR was increased by 10%, the higher luminal flow along the proximal tubule raised transcellular transport via the torque-dependent scaling. That was followed by an increase in paracellular transport, the driving force of which depends on transcellular transport. Consequently, proximal tubule Na^+^ reabsorption was predicted to increase by 11%; that was accompanied by a 10% increase in Cl^−^ reabsorption. Conversely, when SNGFR was reduced by 10%, proximal tubule Na^+^ and Cl^−^ transport decreased by 11 and 10%, respectively. Thus, the model proximal tubule exhibits glomerulotubular balance, albeit not 100%. As SNGFR and Na^+^ delivery to the thick ascending limb increased, luminal [Na^+^] decreased more slowly along this segment. This trend continued through the initial segment of the connecting tubule. With the higher luminal [Na^+^], transcellular Na^+^ reabsorption increased and paracellular secretion decreased. Similar trends were observed for Cl^−^ transport. When SNGFR increased by 10%, urine Na^+^ and Cl^−^ excretion increased by 38 and 95%, respectively; when SNGFR decreased by 10%, urine Na^+^ and Cl^−^ excretion decreasde by 17 and 44%, respectively. Water transport also exhibits similar trends along the water-permeable segments (e.g., proximal tubule, connecting tubule, collecting duct). See Fig 5, panels A and D.

As SNGFR and Na^+^ delivery to the thick ascending limb increased, K^+^ reabsorption via the NKCC2 increased; see Fig 5B. On the other hand, the increased Na^+^ delivery to the connecting tubule and the resulting higher Na^+^ reabsorption yielded substantially higher K^+^ secretion (Fig 5B). Taken together, when SNGFR increased by 10% (decreased by 10%), urine K^+^ excretion increased by 55% (decreased by 33%); see Fig 4B.

### SGLT2 inhibition

The proximal convoluted tubule (a.k.a. S1-S2 segment) expresses the Na^+^-glucose cotransporter 2 (SGLT2) on its apical membrane, whereas the S3 segment expresses SGLT1. Under baseline conditions, the SGLT2 and SGLT1 mediate the uptake of approximately 90 and 10% of the filtered glucose. A new diabetic drug inhibits SGLT2 and thus renal reabsorption of glucose [10], and has been shown to be effective in lowering blood glucose level. Because SGLT2 inhibition affects both Na^+^ and glucose transport, a question is: *To what extent do SGLT2 inhibitors shift Na^+^ transport to downstream nephron segments, and to what extent do these compounds elevate Na^+^ excretion?*

**Fig 6:**
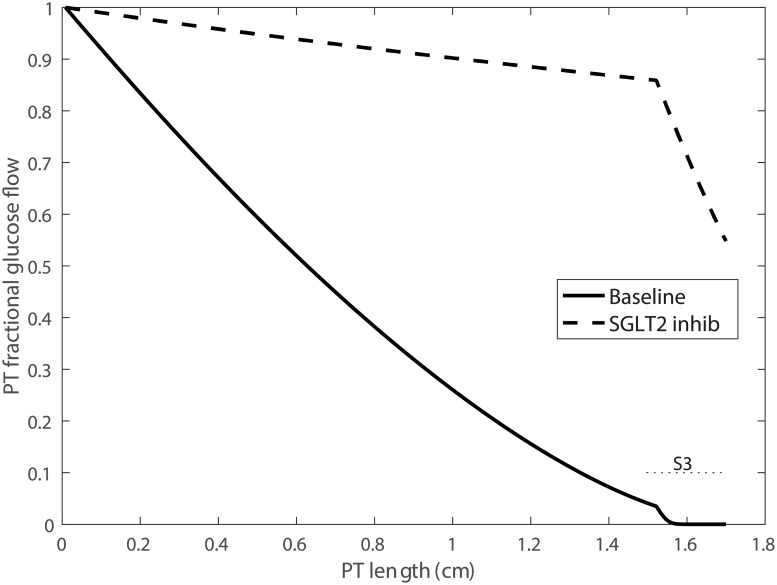
Fractional glucose flow along the proximal tubule (PT), obtained for baseline conditions and with SGLT2 inhibition. Under baseline conditions, the SGLT2 reabsorbed ∼90% of the filtered glucose, with the remaining glucose reabsorbed via the SGLT1 along the S3 segment. With SGLT2 blockade, 56% of the filtered glucose is reabsorbed, primarily via the SGLT1.

To simulate acute SGLT2 blockade, we reduced SNGFR by 3%, based on observations in non-diabetic humans receiving canagliflozin or dapagliflozin for 4 days (personal communication by Volker Vallon). SGLT2 expression was reduced by 90%. These changes resulted in urine glucose excretion of 242 *μ*/min/kidney, which corresponds to a fractional excretion of 54%. Fractional glucose flow along the proximal tubule is shown in Fig 6 for baseline and SGLT2 inhibition. The model predicted that 17% of the filtered glucose was reabsorbed along the S1-S2 segments by the remaining SGLT2 and via the paracellular route, and 29% along the S3 segment across the SGLT1 and the tight junctions.

**Fig 7:**
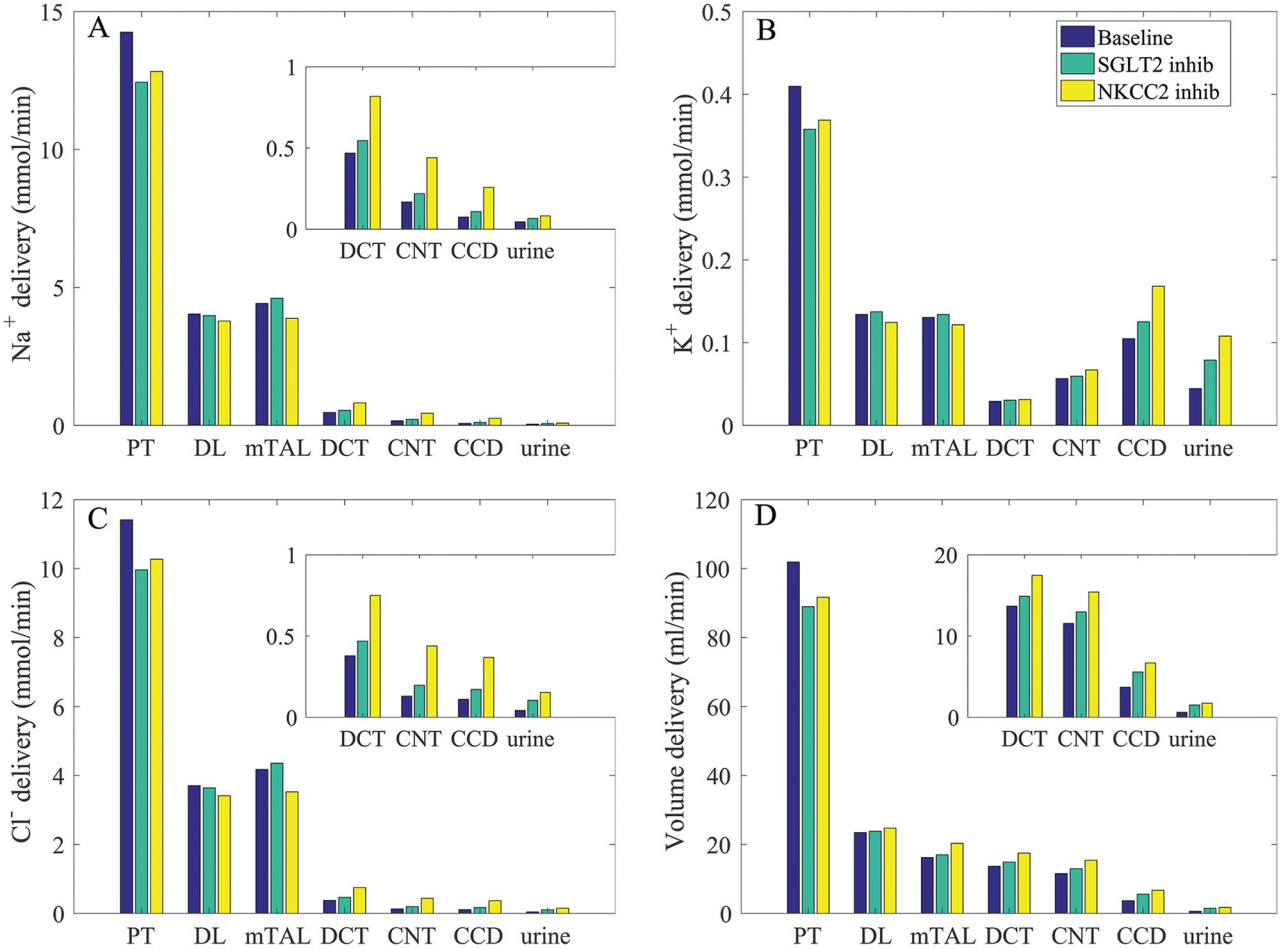
Impact of SGLT2 and NKCC2 inhibition on segmental delivery of Na^+^, K^+^, Cl^−^, and fluid, given per kidney. Notations are analogous to Fig 2. SGLT2 inhibition lowers proximal convoluted tubular transport, whereas NKCC2 inhibition lowers thick ascending limb NaC; transport; both result in diuresis, natriuresis, and kaliuresis. Insets reproduce distal segment and urine values.

The deliveries of Na^+^, K^+^, Cl^−^, and water to individual nephron segments are shown in Fig 7. With SGLT2 inhibition, significantly more glucose was retained in the proximal tubule luminal fluid, thereby increasing its osmolality and inhibiting water reabsorption. In other words, SGLT2 inhibition elicited osmotic diuresis, thereby lowering proximal tubular fluid Na^+^ concentration and reducing passive Na^+^ transport via the paracellular route in that segment. Even though the higher luminal flow conversely stimulated active Na^+^ transport (via torque-induced increases in transcellular transporter expression [11]), the reduction in passive transport was greater. Consequently, net Na^+^ reabsorption decreased in the proximal tubule by 17%. As can be seen in Fig 7, SGLT2 inhibition elevated Na^+^, K^+^, Cl^−^, and volume flow in all nephron segments. Taken together, SGLT2 in euglycemic conditions resulted in diuresis, natriuresis, and kaliuresis, with urine flow, Na^+^ and K^+^ excretion increased by 144, 46, and 77%, respectively (see Table 3, column “SGLT2 inhibition”).

### NKCC2 inhibition

Next, we simulated the effect of a diuretic (furosemide) that may be used in treatment of hypertension. Specifically, we simulated 80% inhibition of NKCC2, the Na^+^-K^+^-2Cl^−^ cotransporter that is expressed on the apical membrane of the thick ascending limbs of the loops of Henle. Inhibiting NKCC2 impairs the kidney’s ability to generate an axial osmolality gradient, with the assumption that the NKCC2 inhibitor was administered for long enough for this washout to occur. Thus, following the approach in our previous study [8], the interstitial fluid concentrations of selected solutes were lowered. Specifically, we assumed that (i) cortical interstitial concentration profiles were unaffected; (ii) the concentrating mechanism of the outer medulla was significantly impaired, so that at the outer-inner medullary boundary, the interstitial concentrations of Na^+^, K^+^, Cl^−^, and urea were reduced to 204, 5.6, 193, and 28 mM, respectively; (iii) at the papillary tip, the interstitial concentrations of Na^+^, K^+^, Cl^−^, and urea were reduced to 160, 7.0, 149, and 56 mM, respectively. With these values, the interstitial fluid osmolality at the papillary tip was 416 mosm/(kg H_2_O).

Segmental delivery of key solutes and fluid is shown in Fig 7. Inhibition of NKCC2 had no direct impact on proximal convoluted tubule transport. However, because medullary interstitial fluid osmolality was assumed to be lower, water reabsorption from the S3 segment and other medullary segments was reduced, resulting in increased volume delivery to downstream segments. Urine flow increased by 2.8 fold, consistent with observations reported in Ref. [36]. See Table 3, column “NKCC2 inhibition.”

**Fig 8:**
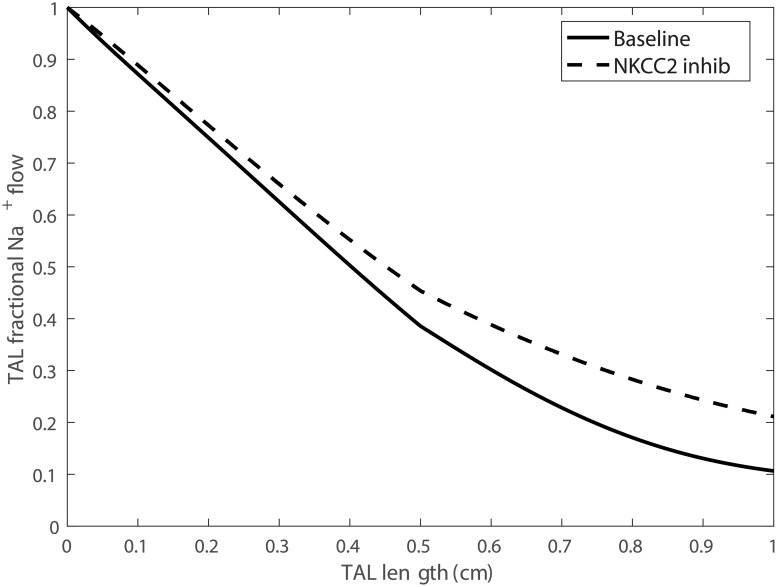
Fractional Na^+^ flow along the thick ascending limb (TAL), obtained for baseline conditions and with NKCC2 inhibition.

Solute delivery to the thick ascending limb was largely minimally affected by 80% NKCC inhibition (see Fig 7, compare “Baseline” and “NKCC2 inhib”). Profiles of fractional Na^+^ flow along the thick ascending limb under baseline conditions and with NKCC2 inhibition are shown in Fig 8. Along the thick ascending limb, Na^+^ transport fell by 20%. That was accompanied by a substantial reduction in the reabsorption of K^+^ and Cl^−^ along the thick ascending limb (results not shown). Consequently, urinary Na^+^, K^+^, and Cl^−^ excretion were predicted to increase by 1.8, 2.4, and 3.6 fold with NKCC2 inhibition.

## Discussion

Our understanding of the kidney has been vastly improved by studies employing techniques that evaluate renal function at the single nephron level. Particularly instrumental and indispensable is the micropuncture technique [37], which has facilitated studies of glomerular filtration and hemodynamics, and tubular epithelial activity in animal models. However, such experiments are considered too invasive to be conducted in the human kidney. To reveal microscopic processes and physiological function in the human kidney, one may utilize functional MRI [38], a non-invasive technique that could facilitate translation of many studies performed in controlled animal models using technologies that are invasive to humans. Another alternative is the computational modeling technique. One notable application of computational models is the simulation of “clean” knockout experiments. Because unlike an animal, a computational knockout model does not need to live, it will not attempt to compensate by adjusting other transport mechanisms.

In this study, we developed the very first computational model of solute and water transport along a human nephron. A number of studies have suggested that a similar set of transporters are expressed along the human and rodent nephrons (e.g., [39–41]). Thus, we based the human nephron model on our published rat model [9, 11]. We first incorporated anatomic and hemodynamic data from humans, and then determined what additional transport parameters need to be adjusted to ensure that model predictions are consistent with known human data. Even though we did not expect a human to be a big rat, a rat model using human anatomic and hemodynamic data, without any change in transport expression levels, generate predictions that are largely (albeit not perfectly) consistent with human data. See Table 3, column “GFR & dimensions.” This result suggests general similarity between the electrophysiology of mammals that survive in similar living environment. With additional adjustments in transporter expression (see below), the model predicts key tubular transport and urine output that are consistent with human values (Table 3, column “Baseline”).

Model simulations were validated by comparing predicted urine output, and possibly other predictions, with measurements in humans. However, it is important to note that the model does not merely recapitulate experimental observations. The model predicts, at every single point along the nephron, luminal fluid flow, luminal solute concentrations, cytosolic solute concentrations, epithelial membrane potential, transcellular solute and water fluxes, and paracellular solute and water fluxes—most of which are virtually impossible to determine in humans *in vivo* under current ethical guidelines. Hence, the model suggests, under various physiological or pharmacological conditions, what transport processes might be taking place within the nephron in order to produce the urine that we observe.

Key model predictions are summarized below:

- Model simulations predict two major differences in transport activity between human and rat: (i) lower SGLT2 density along the human proximal convoluted tubule, and (ii) lower H^+^-ATPase and H^+^-K^+^-ATPase densities along the human collecting duct.
- Under baseline conditions, the model proximal tubule and the thick ascending limb reabsorb most of the filtered Na^+^, K^+^, and Cl^−^; most of the urinary K^+^ is secreted by the distal tubular segments; and the thick ascending limb and, to a lesser extent, the inner-medullary collecting duct acidify the urine.
- The model human proximal tubule exhibits glomerulotubular balance (Fig 5), so that tubular reabsorption of filtered volume and Na^+^ varies with SNGFR, although that balance is not perfect.
- Under physiological conditions the SGLT2 of the model S1-S2 segments and the SGLT1 of the model S3 segment mediate ∼90 and 10% of the filtered glucose, respectively (Fig 6). When SGLT2 is inhibited, the transport capacity of SGLT1 more than doubles so that >40% of the filtered glucose is reabsorbed.
- Inhibition of SGLT2 significantly reduces Na^+^ reabsorption by the model proximal tubule and increases Na^+^ reabsorption by the thick ascending limb (Fig 7).
- Under euglycemic conditions, inhibition of SGLT2 induces significant osmotic diuresis, resulting in natriuresis and kaliuresis; see Table 3, column “SGLT2 inhibition,” and Fig 7.
- NKCC2, which is expressed along the apical membrane of the thick ascending limb, plays an essential role in renal Na^+^ transport. Even an incomplete inhibition of NKCC2 (80%) induces substantial diuresis, natriuresis, and kaliuresis, with urine output elevated several folds (Table 3, column “NKCC2 inhibition,” and Fig 7).

The present model simulates solute and water transport along the nephron of a healthy adult. The model can be used to simulate the nephron of a diabetic patient, if model parameters are appropriately adjusted to capture pathophysiological changes in hemodynamics (to represent diabetes-induced glomerular hyperfiltration), anatomic (tubular hypertrophy), transport and other relevant model parameters (see Table 2 in Ref. [9] or Na^+^ transport expression changes in a diabetic rat). It must be acknowledged that quantifying these pathophysiological changes is a challenging task. Nonetheless, such a model. if successfully formulated, can be used to assess the actions of SGLT2 inhibitors in a diabetic kidney, as was done in a rat [9, 42]. By further adjusting model parameters to simulate a remnant kidney (using our previous approach for the rat [12, 43]), we can simulate the administration of SGLT2 inhibitors to a kidney with diabetic nephropathy. To the extent that renal fluid and Na^+^ excretion can determine blood pressure and heart failure, model results can be used to assess the degree to which cardiovascular benefits SGLT2 inhibitors persist in patients with reduced GFR, and why. To directly predict blood pressure, however, one would need a more comprehensive model such as Ref. [44]. By adjusting SNGFR (to represent changes in renal blood flow regulation) and key transport activity levels (e.g., NHE3 [45]), one can simulate a hypertensive kidney and assess the relative effectiveness of diuretics, and why.

A major motivation for developing a computational model of the human nephron is to to provide a platform for pharmacokinetics and pharmacodynamics simulations; that platform can be applied to predict the effects of a new drug, or to explain the underlying mechanisms of observed effects. In this regard, it is noteworthy that important species differences have been reported in relation to the expression of various membrane transporters that mediate transport of organic anions and cations in the mammalian kidneys and other organs [46]. (Organic anions and cations are not represented in the present model.) A computational model that includes the correct expression of the relevant organic anions/cations (in humans) would prove useful in translating drug test results in rodents to humans.

The present nephron model predicts cellular solute concentrations, tubular flow, and luminal fluid solute concentrations. Except for the proximal tubule, nephron segments are represented as rigid tubules, and cell volume regulation [3] is not represented. Also, SNGFR is assumed known *a priori*. To model autoregulation, SNGFR can be set as a function of downstream tubular flow composition [47–51]. Interstitial fluid composition is assumed known *a priori* (Table 2). Additionally, the model does not represent the vasculature. As a result, the model does not represent the interactions among the nephron segments, or the interactions between nephrons and the renal vasculature. To properly simulate renal handling of a given drug compound, a model that represents the interactions among renal tubule and vessels is needed. Such a model can be formulated by embedding the nephron model into a human renal medullary model (e.g., Ref. [52–54]), similar to the recent rat kidney model by Weinstein [55]. Indeed, the present model can be used as an essential component in an integrated model of kidney function in humans for studying clinically relevant questions such as K^+^-induced natriuresis [55].
